# Cellular Redox Imbalance and Changes of Protein S-glutathionylation Patterns Are Associated with Senescence Induced by Oncogenic H-Ras

**DOI:** 10.1371/journal.pone.0052151

**Published:** 2012-12-20

**Authors:** Tatiana Armeni, Luisa Ercolani, Lorena Urbanelli, Alessandro Magini, Francesca Magherini, Armanda Pugnaloni, Francesco Piva, Alessandra Modesti, Carla Emiliani, Giovanni Principato

**Affiliations:** 1 Department of Clinical Sciences, Section of Biochemistry, Biology and Physics, Università Politecnica delle Marche, Ancona, Italy; 2 Department of Experimental Medicine and Biochemical Sciences, Università di Perugia, Perugia, Italy; 3 Department of Biochemistry, Università di Firenze, Firenze, Italy; 4 Department of Molecular and Clinical Sciences, Università Politecnica delle Marche, Ancona, Italy; Boston University Medical School, United States of America

## Abstract

H-Ras oncogene requires deregulation of additional oncogenes or inactivation of tumor suppressor proteins to increase cell proliferation rate and transform cells. In fact, the expression of the constitutively activated H-RasV12 induces cell growth arrest and premature senescence, which act like barriers in pre-neoplastic lesions. In our experimental model, human fibroblasts transfected with H-RasV12 show a dramatic modification of morphology. H-RasV12 expressing cells also show premature senescence followed by cell death, induced by autophagy and apoptosis. In this context, we provide evidence that in H-RasV12 expressing cells, the premature senescence is associated with cellular redox imbalance as well as with altered post-translation protein modification. In particular, redox imbalance is due to a strong reduction of total antioxidant capacity, and significant decrease of glutathione level. As the reversible addition of glutathione to cysteinyl residues of proteins is an important post-translational regulative modification, we investigated S-glutathionylation in cells expressing active H-Ras. In this contest we observed different S-glutathionylation patterns in control and H-RasV12 expressing cells. Particularly, the GAPDH enzyme showed S-glutathionylation increase and significant enzyme activity depletion in H-Ras V12 cells. In conclusion, we proposed that antioxidant defense reduction, glutathione depletion and subsequent modification of S-glutathionylation of target proteins contribute to arrest cell growth, leading to death of fibroblasts expressing constitutively active H-Ras oncogene, thus acting as oncogenic barriers that obstacle the progression of cell transformation.

## Introduction

Oncogene deregulation is not sufficient to induce cellular proliferation and tumorigenic transformation, which are caused by a variety of cooperating mechanisms. Deregulated oncogenes can increase cellular proliferation rate, but they require additional oncogenes or inactivation of tumour suppressor genes such as p53 or pRb to fully transform cells [1]–[3]. In absence of cooperating mutations, deregulated oncogene activation typically leads to cell cycle arrest, premature senescence and cell death by apoptotosis and autophagy [4]–[7]. These responses act as a tumor suppressor mechanism in the pre-malignant stage of tumorigenesis in order to prevent the progression of oncogenic transformation [8]. Constitutively activated H-RasV12 oncogene induces proliferative arrest and premature senescence in normal fibroblasts (OIS, Oncogene Induced Senescence). These events have been associated with DNA damage and activation of DNA damage response (DDR), which is considered an efficient oncogenic barrier [9]–[10]. Data supporting the activation of DDR by DNA replication stress do not preclude that other types of cell damaging stresses may contribute to OIS and be considered as additional oncogenic barriers [11]–[13]. Furthermore when cells are stimulated to proliferate as in the case of H-RasV12 oncogene expression, the stress due to hyper-proliferation certainly affects many cell structures and not only nuclear DNA.

ROS-mediated cell damage has long been thought to play a role in carcinogenesis initiation and malignant transformation [14], [15]. In fact, many malignant cell types possess an abnormal redox metabolism, which consists in deregulation of antioxidant enzymes, impaired mitochondrial function and enhancement of reactive oxygen species (ROS) production [16]. On the other hand, ROS are considered as second messengers because they may regulate the strength and duration of signalling through redox-dependent signal transduction pathways, via the cyclic oxidation/reduction of cysteine residue in kinase, phosphatases and other regulatory factors [15], [17]. Reactive thiols in proteins are subject to a wide array of irreversible modifications in oxidation state, including oxidation to sulfenic (-SOH), sulfinic (-SO_2_H), and sulfonic (-SO_3_) acid and formation of disulfide bridges. Since these over-oxidation reactions are by nature irreversible, the thiol modifications usually play only a minor role in controlling redox–regulated proteins [18], whereas changes in the reversible oxidation state of cysteine residues, such as nitrosylation and glutathionylation, are important post-translational protein modifications with a critical role in signal transduction. Protein S-glutathionylation (protein-SSG) plays a dual role in cell biology – as an antioxidant because it provides protection of protein cysteines from irreversible oxidation and as signal transduction mechanism [19], [20]. *S*-glutathionylation is an important mechanism for dynamic post-translational regulation of a variety of regulatory, structural and metabolic proteins [21], [22]. In particular, signalling proteins (especially kinases and phosphatases), cytoskeleton proteins, proteins involved in metabolism and energy, folding proteins and redox homeostasis protein appear to be regulated by *S*-glutathionylation [23]. *S*-glutathionylation consists in the reversible formation of mixed disulfides between glutathione and protein cysteinyl residues of proteins and has a critical role in sulfhydryl homeostasis. Since glutathione (GSH) is considered as a thiol redox buffer, S-glutathionylation can be directly linked to the redox status of cell GSH [24], [25]. Interestingly, it has been reported that S-glutathionylation may modulate the activity of oncogenes such as Ras or transcription factors like p53 [26], [27] and also that GSH is involved in regulating the activation of various key proteins regulating cell proliferation, including nuclear factor NF-kB and activator protein AP-1. [28]–[30].

In various types of tumor cells the high content of GSH generally increases antioxidant capacity and resistance to oxidative stress, and makes cancer cells chemo-resistant. Although neither the mechanism nor the implications of these changes are well defined, agents that deplete GSH, such as buthionine sulfoximine, have shown clinical anti-cancer activity; thus is it possible to suppose that the high GSH content may be necessary for cancer cell survival [31]–[33]. To gain insight the mechanism of Ras-initiated cell death and the role of GSH in pre-malignant cells model, we used human fibroblasts expressing constitutively active H-RasV12 and evaluated redox balance, GSH:GSSG ratio and protein *S*-glutathionylation level and pattern. In this study, we demonstrate that expressing constitutively active H-Ras oncogene induced cell cycle progression and that the cells activated the pathways for induced premature senescence followed by autophagy and apoptosis. During the senescence state we observed cell redox imbalance that involved antioxidant defense reduction, glutathione depletion and modification of S-glutathionylation pattern. Finally, our study brings a better understanding to the role of redox cell system in OIS.

## Materials and Methods

### Reagents

All cell culture reagents were obtained by PAA the cell culture company (Pasching, Austria). All chemical reagents were obtained by Sigma Aldrich (Sigma, St Louis, MO, USA). Lipofectamine LTX with expression vector pcDNA™6/myc-His and Blasticidin-S, Annexin V-FITC Apoptosis Detection kit and carboxy-H_2_DCFDA (C400) were supplied by Invitrogen (Invitrogen, Carlsbad, CA, USA), “In situ cell proliferation kit FLUOS” was supplied by Roche (Roche Diagnostics GmbH, Mannheim, Germany), mouse monoclonal anti-glutathione antibody and rabbit polyclonal anti-H Ras antibody were supplied by Santa Cruz Biotechnology (Santa Cruz Biotechnology, CA, USA), and Immobiline™DryStrip pH 3–10 non linear supplied by GE Healthcare. pSG5-RasV12, was kindly provided by Dr. Giuliana Pelicci, Department of Experimental Oncology, European Institute of Oncology, Milan, Italy.

### Cell Culture and Growth Curve

HuDE (human dermal fibroblasts) were purchased from the Istituto Zooprofilattico Sperimentale, Brescia, Italy. The cells were cultured in DMEM medium supplemented with 10% (v/v) heat inactivated fetal calf serum, 2 mM glutamine, 10 U/ml penicillin, 10 µg/ml streptomycin at 37°C in a humidified atmosphere containing 5% (v/v) CO_2_. Cell growth was determined by counting cell numbers in a haemocytometer. The viability of the cells was estimated by examining their ability to exclude Trypan blue (0.1% in 0.9% NaCl).

### pcDNA6 Vectors and H-Ras Mutant Expression

Cells were transfected by lipofection using Lipofectamine LTX with the expression vector pcDNA™6/myc-His encoding the constitutively active mutant H-RasV12. This mutation involves the replacement of the amino acid glycine with a valine, which makes the GTPase constitutively GTP bound. The vector expressing the Ras mutant was obtained by subcloning H-RasV12 from pSG5 vector into the EcoRI site of pcDNA™6/myc-His. Transiently transfected fibroblasts were selected using 4µg/ml Blasticidin-S for 5 days.

### Cellular Extracts

Cell samples were washed twice with 0.9% NaCl (500 g for 10 minutes in top centrifuge) and suspended in 10 mM sodium phosphate pH 6.0 buffer. Cellular suspensions were lysed by 5 freeze (in liquid nitrogen) and thaw (room temperature) cycles. Total cells disruption was validated using trypan blue staining. Lysates were centrifuged at 16,000 g (20 minutes, 4°C). Extracts for immunoblotting were obtained by treating cells with 10 mM sodium phosphate pH 6.0 buffer and 0.5% (v/v) Nonidet P40 detergent. After 1 h incubation on ice, they were centrifuged at 16,000 g for 20 minutes. All procedures were carried out at 4°C. Supernatants were recovered and the protein content was determined by the method of Bradford [34] using BSA as standard.

### Cell Cycle Analysis

All cells were analyzed using a Beckman Coulter Epics XL flow cytometer and all measurements were performed under the same instrument setting. FACS DNA content analysis was carried out by propidium iodide method. Cells were harvested and washed twice with cold PBS and fixed with 70% ice cold ethanol for 2 h at 4°C. The fixed cells were suspended in a master mix solution containing PBS, propidium iodide (final concentration: 40 µg/ml) and DNase-free RNaseA (final concentration: 100 µg/ml) for 30 minutes at room temperature in the dark. At least 10,000 cells for each sample were measured. The percentage of cell cycle phases (G0/G1, S, and G2/M) was quantified by MultiCycle AV DNA analysis plug-in for FCS Express (De Novo Software, CA, USA). Control and H-RasV12 cells were seeded in 6-multiwell after selection with basticidin-S, in order to detect S-phase cells and determine cell cycle kinetics. Cells were treated with 40 µM bromodeoxyuridine (BrdU) for 30 minutes at 37°C and grown in fresh medium for 3, 6, 18 and 24 h, then fixed O/N in 70% ethanol. Immunostaining and flow cytometry analyses were carried out with “In situ cell proliferation kit FLUOS”, according to the manufacturer’s instructions. BrdU untreated cells were used as negative control at each time point. At least 10,000 cells for each sample were measured for BrdU positive staining. The percentage of BrdU positive cells was quantified subtracting the negative control at any time point.

### Apoptosis Analysis

Apoptosis was analyzed by flow cytometry, using Annexin V-FITC Apoptosis Detection kit, according to the manufacturer’s instructions. Briefly, control and H-RasV12 cells were trypsinized, washed twice with cold PBS and resuspended in 1X Binding Buffer at final concentration of 1,0×10^6^ cell/ml. Annexin V-FITC (0.25 µg/ml) and Propidium Iodide (PI) (1 µg/ml) were added to cell suspension and incubated for 10 minutes at room temperature, protected from light. Samples were analyzed using Beckman Coulter EPICS XL flow cytometer. For each sample 10,000 events were acquired. Annexin V-FITC is detected as a green fluorescence and propidium iodide is detected as a red fluorescence. Early apoptosis is defined by Annexin V^+^/PI^−^ staining, late apoptosis is defined by Annexin V^+^/PI^+^ staining and necrosis is defined by Annexin V−/PI+ staining.

### Senescence-associated β-galactosidase Staining

Senescent cells were evaluated by SA-β-galactosidase (SA-β-gal) staining as described previously [35]. Control and H-RasV12 transfected cells were seeded in 6-multiwell plates. 24 h after the seeding, cells were washed once with phosphate buffer saline (PBS) and then fixed with 0.5% glutaraldehyde in PBS for 15 minutes at room temperature. Plates were washed twice with PBS. Cells were incubated at 37°C overnight with the staining solution (5 mM potassium ferrocyanide, 5 mM potassium ferricyanide, 2 mM MgCl_2_ in 100 mM citric acid and 200 mM Na_2_HPO_4_ solutions pH 6.0, 1 mg/ml 5-bromo-4-chloro-3-indolyl β-D-galactopyranoside). Blue color development was checked under microscope using 60× total magnification. Positive fibroblasts were counted and results were expressed as mean ± s.e.m. of the percentage of SA-β-gal positive fibroblasts with respect to the total number of fibroblasts.

### Reactive Oxygen Species (ROS) Detection

Intracellular ROS levels were detected by flow cytometry using carboxy-H_2_DCFDA (C400), as probe, according to the manufacturer’s instructions. Within cells, carboxy-H_2_DCFDA is hydrolyzed by esterase to form a non-fluorescent polar derivative, which is oxidized by intracellular ROS to form the fluorescent compound 2′,7′-dichlorofluorescein (DCF), whose emission maximum is at 520 nm. Control and H-RasV12 cells were trypsinized, washed twice with cold PBS and suspended at a final concentration of 0,5×10^6^ cell/ml in prewarmed PBS containing the probe at a working concentration of 10 µM, then incubated for 30 minutes. As positive control, cells treated with 250 µM of H_2_O_2_ for 20 minutes on ice were used. After incubation, cells were washed twice in PBS and labelled with 20 µg/ml PI, in order to remove false negative results due to cells with compromised plasma membrane. Fluorescence of labelled cells was measured on a Beckman Coulter EPICS XL flow cytometer using an excitation wavelength of 488 nm. For each sample, the fluorescence intensity of 10,000 negative PI cells was recorded.

### Total Antioxidant Capacity

Total antioxidant capacity for peroxyl radicals was evaluated using TOSC assay, which measures the ability of cellular antioxidants to absorb oxyradicals artificially generated by 2-2′-azo-bis-(2 methylpropionamidine)-dihydrochloride (ABAP), a reaction inhibiting the oxidation of α-keto-γ-methiolbutyric acid (KMBA) to ethylene. Reactions were conducted using 50 µl of cell extracts and 2 mM KMBA, 20 mM ABAP in 100 mM potassium phosphate buffer, pH 7.4, as previously reported [36]. TOSC values were quantified using the equation: TOSC = 100−(SA/CA×100), where SA is the integrated area calculated under the least squares kinetic curve produced during the sample reaction and CA is the integrated area calculated under the least squares kinetic curve produced during the reaction for the control. For all measurements, TOSC values were referred to the protein concentration of each sample.

### Biochemical Analysis

Total glutathione (GSH+GSSG), oxidize glutathione (GSSG) and total thiol groups were measured spectrophotometrically by the glutathione reductase (GR) recycling assay at 412 nm in the presence of 5,5′-dithiobis(2-nitrobenzoic) acid (DTNB) [37]. Briefly, the absorbance at 412 nm generated by DTNB was measured and then GSH and GSSG concentration were calculated using a calibration line obtained with known concentrations of GSH and GSSG. For sample preparation, transfected cells were trypsinized and washed twice in cold PBS. For total GSH/GSSG determination, after cell centrifugation, the pellet was resuspended with 1% sulfosalicilic acid, vortexed and then incubated 30 minutes at 4°C. Samples were then centrifuged for 2 minutes at 2,300 g. Supernatant was recovered and divided in two aliquots: one was conserved at 4°C for GSH quantification, the other one was treated with 2-vinilpiridin (C_f_ = 5%) and triethanolamine 20% (C_f_ = 1%) to mask the GSH present in the extract and prevent its measurement. Pellet was resuspended with 1 M NaOH for recovery and quantification of proteins. Total thiol groups were analyzed on total cell extracts.

Glutathione reductase (GR) activity was measured using the method described by Carlberg and Mannervik [38].The assay evaluates the decrease in absorbance at 340 nm due to NADPH oxidation during the reduction of GSSG (ε = −6.22 mM^−1^ cm^−1^). The assay was carried out in 100 mM sodium phosphate pH 7.0 buffer, 0.1 mM NADPH and 1 mM GSSG. The activity of GR was calculated using an extinction coefficient for NADPH of 6.22 mM^−1^•cm^−1^ and the results were expressed as µmol of NADP^+^ per minute per mg of proteins.

Glutahione-S-transferase (GST) activity was measured following the method described by Habig and colleagues [39], using 1-chloro-2,4-dinitrobenzene (CDNB) as substrate at 340 nm. The assay was carried out in 100 mM sodium phosphate pH 6.5 buffer, 1 mM CDNB and 1 mM GSH. GST activity, defined as the amount of enzyme producing 1 µmol of CDNB-GSH conjugate/min under the conditions of the assay, was calculated using an extinction coefficient for CDNB of 9.6 mM^−1^•cm^−1^. Results were expressed as µmol of CDNB-GSH conjugates per minute per mg of proteins.

Glyceraldehyde 3-phosphate dehydrogenase (GAPDH) activity was measured spectrophotometrically at 25°C by monitoring the decrease in absorbance of NADH for 1 min at 340 nm [40]. The reaction mixture contained 0.1 M potassium phosphate buffer, pH 7.6, 6 mM glycerate 3-phosphate, 1.1 mM ATP, 0.9 mM EDTA, 1.7 mM MgSO_4_, 0.02 mM NADH, 14.8 units/ml phosphoglycerate kinase (E.C.2.7.2.3). Results were expressed as µmol of GAPDH per minute per mg of proteins.

### Morphology Analysis

Pellets were fixed in 2% (v/v) glutaraldehyde in 0.1 M sodium cacodylate buffer (pH 7.4) for 1 h at 4°C, washed in the same buffer added with 7% sucrose and post-fixed in 1% OsO_4_ in 0.1 M sodium cacodylate buffer for 1 h at 4°C. Pellets were then washed three times in 0.1 M sodium cacodylate buffer, dehydrated with a graded alcohol series (70%, 95%, and 100%) and propylene oxide, then embedded in araldite resin and polymerized for 48 h at 60°C. Semi-thin sections stained with toluidine blue were examined with a light microscope (Eclipse E 600, Nikon, Italy). Images were recorded with a Nikon Coolpix digital camera (Nikon, Italia). Ultrathin sections were cut with an LKB NOVA ultratome (Bromma, Sweden), placed onto copper grids and stained with 1% (w/v) uranyl acetate in alcohol solution and lead citrate. Ultrastructural analysis and photomicroscopy were performed with a CM 12 transmission electron microscope (Philips; Endhoven, the Netherlands) at 100 kV. Images were recorded by an SIS Mega View III TEM CCD Camera (Olympus; Münster, Germany).

### Immunoblotting

Cell proteins (30 µg) were electrophoresed on 10% acrylamide/bis-acrylamide gel at 150 V for 1 h. Proteins were transferred to PVDF membrane at 100 V for 1 h. Membrane was blocked by 5% non-fat dry milk in Tris-buffered saline with 0.1% Tween 20 (TBST) at room temperature for 1 h. Mouse monoclonal anti-GSH antibody and rabbit polyclonal anti-HRas antibody were used to analyze the S-glutathionylation levels in control and H-RasV12 transfected samples. As negative control, samples were treated with 25 mM DTT. As internal control, membranes were probed with mouse monoclonal anti β-actin. In Dot Blot analysis 3 µg of protein extract were serially diluted 1∶2 till 1∶256 and spotted into a nitrocellulose membrane. Goat anti-mouse and goat anti-rabbit HRP-linked secondary antibodies were used according to manufacturer’s instructions. Immunoblots were detected by chemiluminescence using ECL (GE Healthcare Uppsala, Sweden).

### 2-D Electrophoresis

Control and H-RasV12 transfected cells were harvested and resuspended with 8 M urea and 4% CHAPS to allow the recovery of total protein extracts, then quantified by Bradford assay. First dimension (isoelectrofocusing, IEF) was performed using a 7 cm Immobiline™DryStrip pH 3–10 non linear. Strips were actively rehydrated in the presence of 100 µg of each sample at 30 V for 12 h in an IPG-phor system (Amersham Pharmacia, Amersham, UK). After rehydration, IEF runs were carried out at 20°C based on current limit of 50 *µ*A/IPG-strip as follows: 300 V for 1 h, 3000V for 3 h (gradient) and 3000 V until 15000 V/h was reached in total. After IEF, IPG-strips were equilibrated in a buffer containing 6 M urea, 30% (v/v) glycerol, 2% (w/v) SDS, 50 mM Tris-HCl pH 8.8 for 30 minutes. SDS-PAGE (second dimension) was performed using 10% acrylamide/bis-acrylamide gel and carried out at 90 V for 130 minutes. First and second dimensions were carried out in non reducing conditions to avoid bonds disruption between protein free cysteinyl groups and GSH. Gels were then blotted on PVDF membrane by semi-dry method at 100 mA (0.8 mA/cm^2^) for 75 minutes. A total protein loading control was performed loading 20 µg of the same extracts used for 2-D electrophoresis in 10% acrylamide/bis-acrylamide gel. At the end gel was staining with Coomassie Blue and total protein intensity was evaluated.

### Mass Spectrometry Analysis

Protein identification was carried out by peptide mass fingerprinting (PMF) on Ettan MALDI-TOF Pro mass spectometer (GE Healthcare) as previously described [41]. After visualization by colloidal Comassie staining protocol, spots were mechanically excised, destained in 2.5 mM ammonium bicarbonate and 50% (v/v) acetonitrile and finally dehydrated in acetonitrile. They were then rehydratated in trypsin solution and in-gel protein digestion was performed by overnight incubation at 37°C. Each protein digest (0.75 µL) was spotted onto the MALDI target and allowed to air dry. Then 0.75 µL of matrix solution (saturated solution of α-cyano-4-hydroxycinnamic acid in 50% (v/v) acetonitrile and 0.5% (v/v) TFA) was applied to the sample which was dried again. Mass spectra were acquired automatically using the Ettan MALDI Evaluation software (GE Healthcare). Recorded spectra were calibrated using, as internal standard, the 842.509 and 2211.105 m/z peptides arising from trypsin autoproteolysis. The resulting mass lists were filtered for contaminant removal: mass matrix-related ions, trypsin auto-lysis and keratin peaks. Protein identification by Peptide Mass Fingerprints search was performed using MASCOT version 2.2 as the search engine (Matrix Science, London, UK, http://www.matrixscience.com) with the Swiss-Prot/UniprotKB database. Taxonomy was limited to *Homo sapiens*, a mass tolerance of 100 ppm was allowed and the number of accepted missed cleavage sites was set to one. Alkylation of cysteine by carbamidomethylation was considered as a fixed modification, while oxidation of methionine was considered as a possible modification. The criteria used to accept identifications included the extent of sequence coverage (at least 10%), the number of matched peptides (at least 5) and a probabilistic score at p<0.05.

### Treatment of the H-RasV12 Expressing Cells with Antioxidants

At the end of selection H-RasV12 expressing cells were treated with an antioxidants mix (0.5 mM GSH ethyl ester, 50 µM α-tocopherol and 1.5 mM vitamin C). The treatment was repeated every 24 hours. Cell growth was determined by counting cell numbers in a haemocytometer. The viability of the cells was estimated by examining their ability to exclude Trypan blue (0.1% in 0.9% NaCl).

### Statistical Analysis

Data were presented as mean ± s.d or mean ± s.e.m. Statistical comparison of differences among groups of data was carried out using a Student’s t-test. P-values ≤0.05 were considered statistically significant and P-values ≤0.01 were considered highly significant.

## Results

### Oncogenic H-RasV12 Expression Results in no Increase in Cell Population and Arrest of Cell Cycle

**Figure 1:**
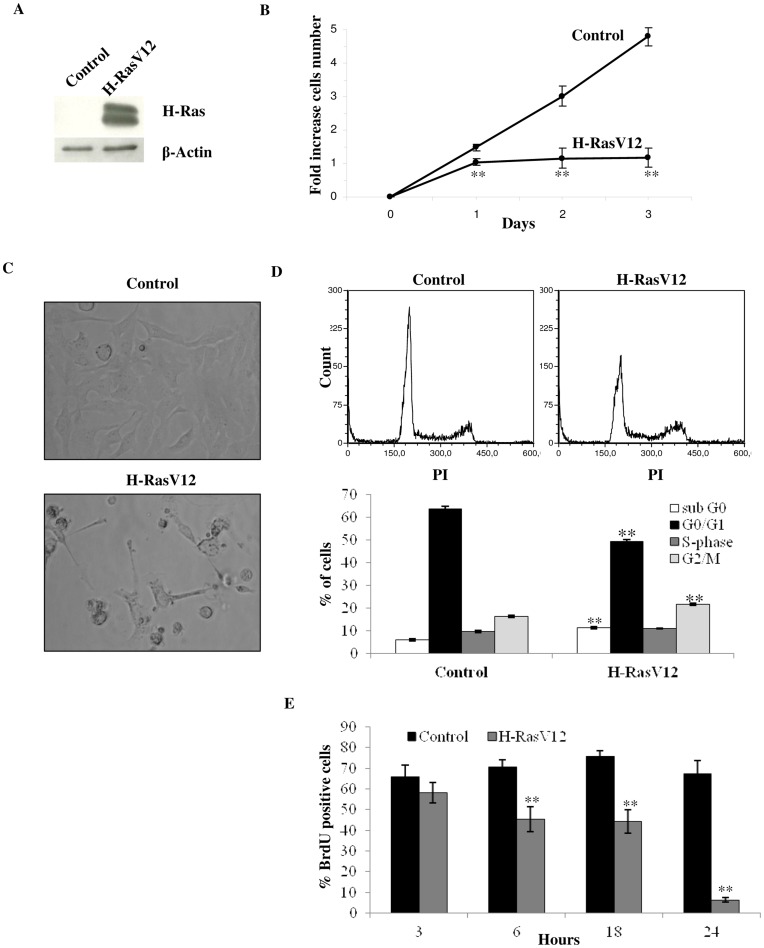
Analysis of cell growth and cell cycle. A) Immunoblotting analysis of H-RasV12 transfected cells. Cell extracts were incubated with an anti-H-Ras antibody. As internal control an anti-β actin antibody was used. The increased expression of H-Ras in transfected cells compared to empty vector transfected cells (control) is shown. B) Growth curve of HuDe fibroblasts transfected with empty vector and H-RasV12. The cell number increase after the end of the selection with blasticidin-S (4 µg/ml) is reported. Mean values were calculated on 5 replicates and mean ± s.d. was indicated for each sample. C) Cell morphology analysis by light microscopy, 200× total magnification. D) Flow cytometry cell cycle analysis of transfected cells using PI (40 µg/ml) as probe. Percentage of fluorescent cells for sub G0, G0/G1, S and G2/M phases were reported in the bottom panel. Mean ± s.e.m. of three analyses is reported. E) Cell proliferation measured by BrdU incorporation in newly synthesized DNA using immunostaining and flow citometry analysis. Control and H-RasV12 transfected cells were treated with 40 µM BrdU for 30 minutes and analyzed after 3, 6, 18 and 24 h. Cells without BrdU were used as negative control according to the manufacture’s instruction. Values of the BrdU positive cells are given as mean ± s.e.m. of three independent experiments subtracting the relative negative control. **P<0.01.

Human fibroblasts (HuDe) were transfected with a pcDNA6 plasmid coding for H-RasV12, a mutant of the effector domain of H-Ras which is constitutively activated, as the mutation makes the GTPase unable to hydrolyze GTP. HuDe were also transfected with the pcDNA6 vector alone as control, and very high levels of Ras expression were detected by immunoblotting (Fig. 1A). Expression of the constitutively activated oncogene H-RasV12 has been previously reported to lead to an arrest of cell proliferation in 1–3 days from clone selection [10]. Microscope analysis of cells transfected with H-RasV12 showed a senescent phenotype within one day from the end of the selection, with a flattened cellular morphology, followed by detachment from the adhesion surface (Fig. 1C). Besides, H-RasV12 transfected cells showed a cell growth arrest that was already evident one day after the end of the selection, compared to cells transfected with the empty vector alone as control (Fig. 1B). Interestingly, analysis by flow cytometry showed that significant fractions of H-RasV12 expressing cells are still in G0/G1 and S-phase of the cell cycle (Fig. 1D), despite their arrested state and altered morphology. Indeed, cell cycle analysis showed that transfected cells had a 14.3% reduction of G0/G1 phase cells and a 6.4% increase of G2/M phase cells, in comparison with control cells. Moreover a 5.2% increase of cells in sub-G0 phase was present in H-RasV12 expressing cells compared to the control, suggesting a possible apoptosis induction (Fig. 1D). BrdU analysis showed a meaningful decrease in BrdU incorporation in H-RasV12 cells after 24 h from treatment (Fig. 1E), indicating an arrest of cell cycle. Indeed, while control cells tranfected with empty vector showed about 70% of positivity to BrdU, which remains constant over 24 h, H-RasV12 cells showed a drop off in BrdU positivity from about 60% after 3 h to 6% after 24 h.

### Oncogenic H-RasV12 Promotes Premature Senescence followed by Cell Death through Autophagy and Apoptosis

**Figure 2:**
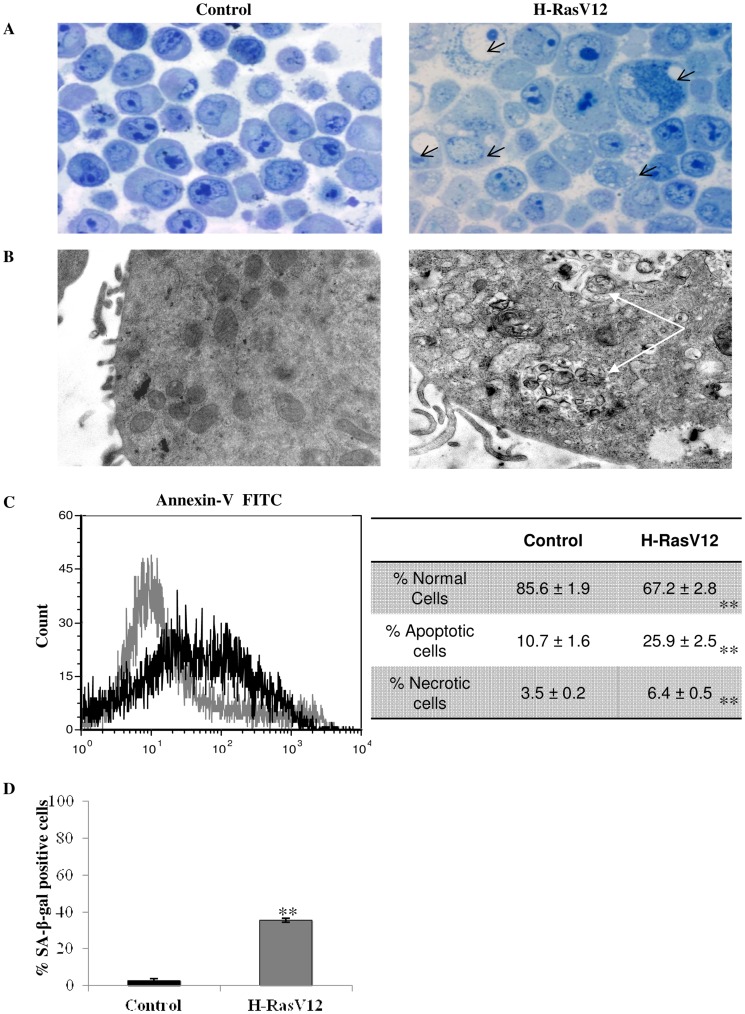
Effect of H-RasV12 on cell vitality. A) Semi-thin sections of control and H-RasV12 cells obtained by light micrographs, 40× original magnification. Black arrows indicate the presence of vacuolization. B) Ultrastructural analysis of control (left photo) and H-RasV12 cells (right photo) obtained by transmission electron microscope (Philips; Endhoven, the Netherlands) at 100 kV. White arrows indicate macro-vacuolization and undigested material. C) Annexin-V FITC flow cytometry analysis for apoptosis. Control cells are indicated in grey, H-RasV12 transfected cells are indicated in black. In the table results are the mean ± s.e.m of percentage values for normal, apoptotic and necrotic cells in three independent experiments. D) Senescence-associated β-galactosidase staining of control and H-RasV12 cells. Percentages of SA-β-gal positive cells were counted at least on five different fields in three independent experiments. **P<0.01.

Oncogenic H-RasV12 has been previously described to induce cell death by different mechanisms, such as premature senescence, apoptosis and autophagy [5]. The morphology of H-RasV12 expressing cells is dramatically different when compared with that of cells transfected with the vector alone as control. In particular morphological semi-thin sections (Fig. 2A) showed a wide cytosolic vacuolization in H-RasV12 expressing fibroblasts, which is usually considered as evidence that cell death is induced as a result of prolonged autophagy [4]. Indeed electron microscopy showed numerous vacuoles with undigested material and mitochondrial autophagy in H-RasV12 expressing cells that indicated macroautophagy processes (Fig. 2B). Besides some nuclei showed apoptotic chromatin and the annexin V-FITC analysis confirmed a higher percentage of phosphatidylserine out of the cell membrane (+15%) in H-RasV12 expressing cells, thus suggesting the induction of apoptosis (Fig. 2C). To assess the induction of premature senescence, we measured the activity of senescence-associated β-galactosidase activity (SA-β-gal), a lysosomal enzyme that is a well known marker of senescence [42]. Results confirmed that many H-RasV12 expressing cells showed premature aging within one day from the transfection (Fig. 2D). All this evidence taken together confirmed that expression of constitutively active H-Ras oncogene promotes premature senescence followed by cell death.

### Constitutively Activated Oncogene H-Ras Induces Cellular Redox Imbalance

**Figure 3:**
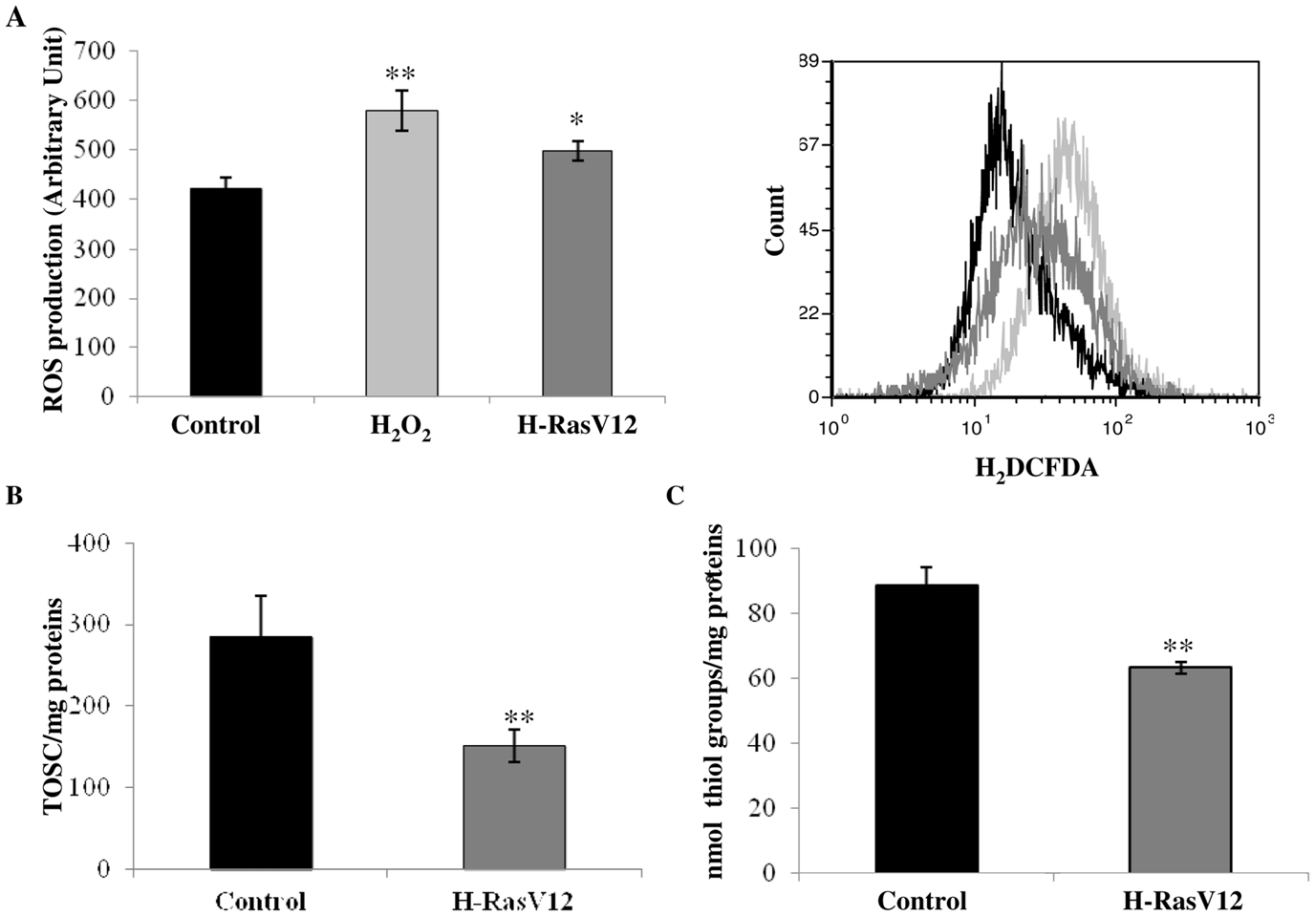
Analysis of cell redox state. A) Measurement of ROS production. Carboxy-H2DCFDA (C400) probe was used to analyze ROS concentration in control and H-RasV12 transfected cells by flow cytometry. In the left panel % of ROS production was reported as fluorescence arbitrary units, obtained by subtraction of PI to carboxy-H2DCFDA. In the right panel cytometric graphs were reported for control cells (black), H-RasV12 cells (dark grey), in comparison with positive control cells treated with 250 µM H_2_O_2_ (light grey). B) Total antioxidant capacity for control and H-RasV12 transfected cells. Results reported are the mean values ± s.d. of three different experiments. C) Quantification of thiol groups which are present in total protein fraction. *P<0.05; **P<0.01.

**Figure 4:**
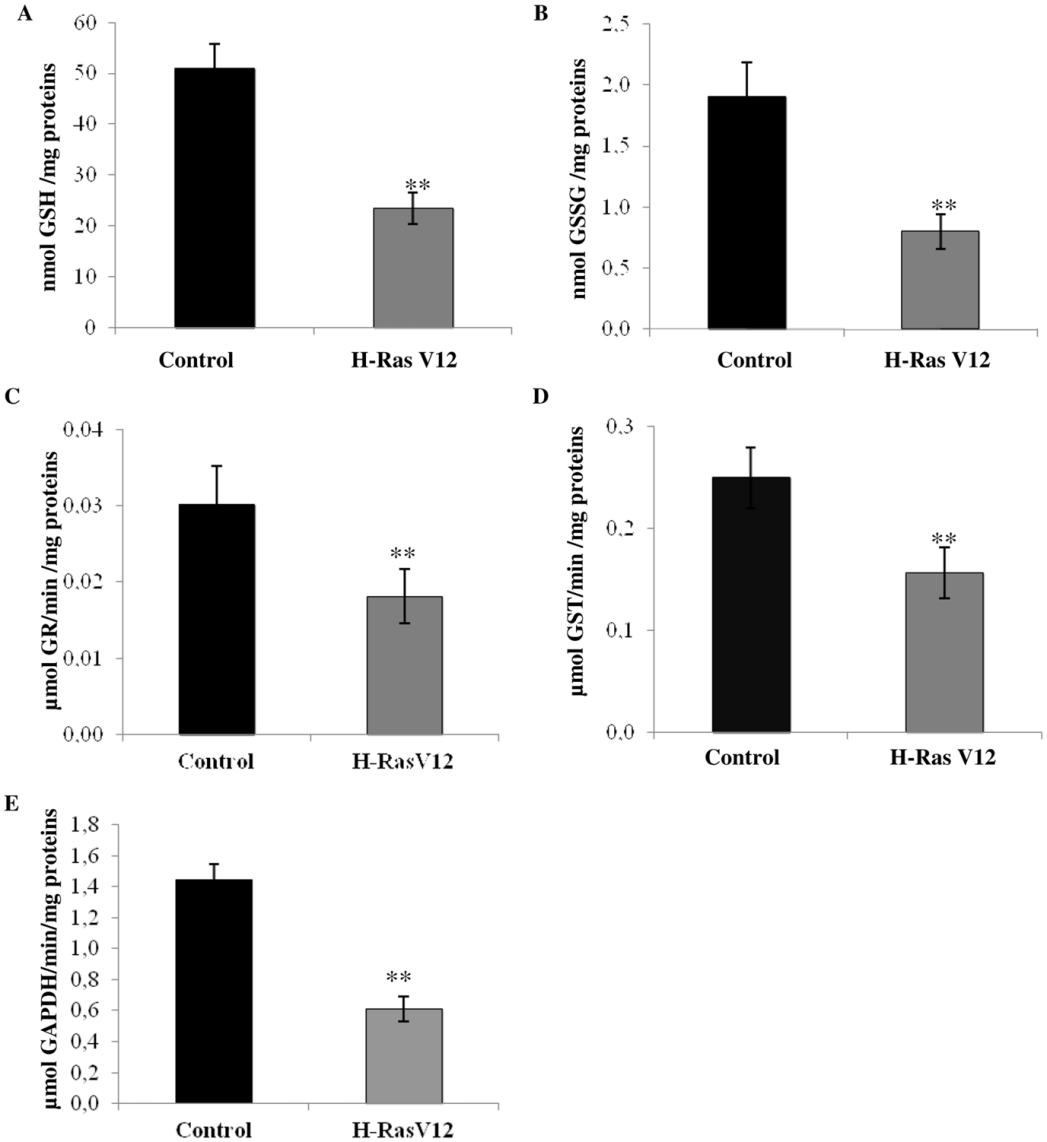
Measurement of glutathione concentration, glutathione dependent enzymes and GAPDH. Total cellular GSH (A) and GSSG (B) were determined for control and H-RasV12 cells by DTNB-GSSG reductase recycling assay. The amount of GSH or GSSG was normalized for proteins content. Values are the mean ± s.d. of six different experiments. Enzyme activity of C) glutathione reductase, D) glutathione-S-transferase and E) glyceraldehyde 3-phosphate dehydrogenase was determined spectrophotometrically at 340 nm. Results reported are mean values ± s.d. of six different experiments. *P<0.05; **P<0.01.

To identify other mechanisms possibly acting as oncogenic barriers during OIS, we evaluated cellular redox balance assessing both reactive ROS production and total antioxidant capacity in cells expressing with H-RasV12, compared with control cells. ROS concentration was significantly higher in H-RasV12 cells with respect to the control but was lower compared to the control cells treated with H_2_O_2_ (Fig. 3A). Also we observed a strong significant depletion of total antioxidant capacity in H-RasV12 transfected cells, as demonstrated by the measurement of cell antioxidant defence against peroxyl radical (Fig. 3B) and significant depletion of total thiol groups (Fig. 3C). Since GSH is the main and most abundant anti-oxidant molecule in the cell, we supposed that depletion of total antioxidant capacity could be due primarily to the reduction of total GSH concentration. In fact concentration of total GSH was significantly lower in H-RasV12 expressing cells compared to control cells, and GSSG also showed to be depleted in H-RasV12 transfected cells (Fig. 4 A,B). As cells transfected with H-RasV12 showed depletion of both total GSH and oxidized GSSG, the GSH:GSSG ratio remained unchanged (control cells 26,9±1.3 and H-RasV12 cells 28,8±1.5). We also determined the activity of two enzymes involved in GSH metabolism and observed that the activity of the GR and GST showed a significant decrease in H-RasV12 cells compared with control cells (Fig. 4 C,D). Total GSH is known to be used by cells for many purposes but the unchanged GSH:GSSG ratio may indicate that the cells can utilizes both GSH and GSSG and both forms of glutathione could be used to bind free sulphydryl groups of proteins to cysteine. Moreover, previous study showed a relationship between senescence and GAPDH activity and we chose this enzyme to evaluate its activity in relationship with oncogene H-Ras expression. We observed a significant depletion of GAPDH activity in H-RasV12 transfected cells (Fig. 4E) compared to the control cells.

### Constitutively Activated Oncogene H-Ras Modifies Protein S-glutathionylation

**Figure 5:**
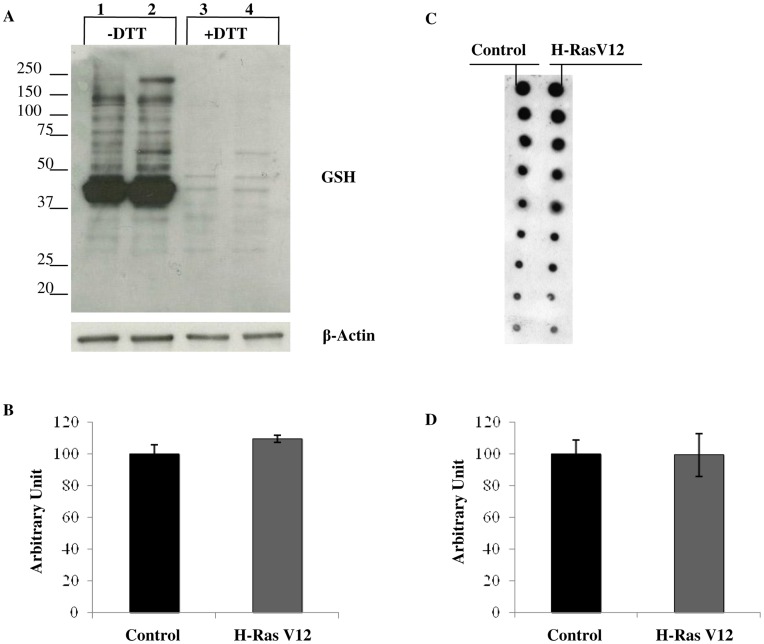
Analysis of protein S-glutathionylation. A) Immunoblotting with anti-GSH antibody of control (lanes 1, 3) and H-RasV12 expressing cells (lanes 2, 4). Proteins (30 µg) were electrophoresed on 10% acrylamide/bis-acrylamide gel. Cell extracts were obtained either in non-reducing (lanes 1, 2) or in reducing (lanes 3, 4) conditions, by means of 25 mM DTT addition prior to loading. An anti-β actin antibody was used as internal control. B) Densitometric analysis of total bands intensity of immunoblotting was reported. No statistical difference of total glutathionylated proteins were observed in H-RasV12 expressing cells in comparison with the control cells C) Dot blot analysis was reported. 3µg of protein extract were serially diluted 1∶2 till 1∶256, spotted into a nitrocellulose membrane and immunoblotted with anti GSH antibody. D) Densitometric analysis of dot blot showed no statistical difference in H-RasV12 expressing cells in comparison with the control cells.

**Figure 6:**
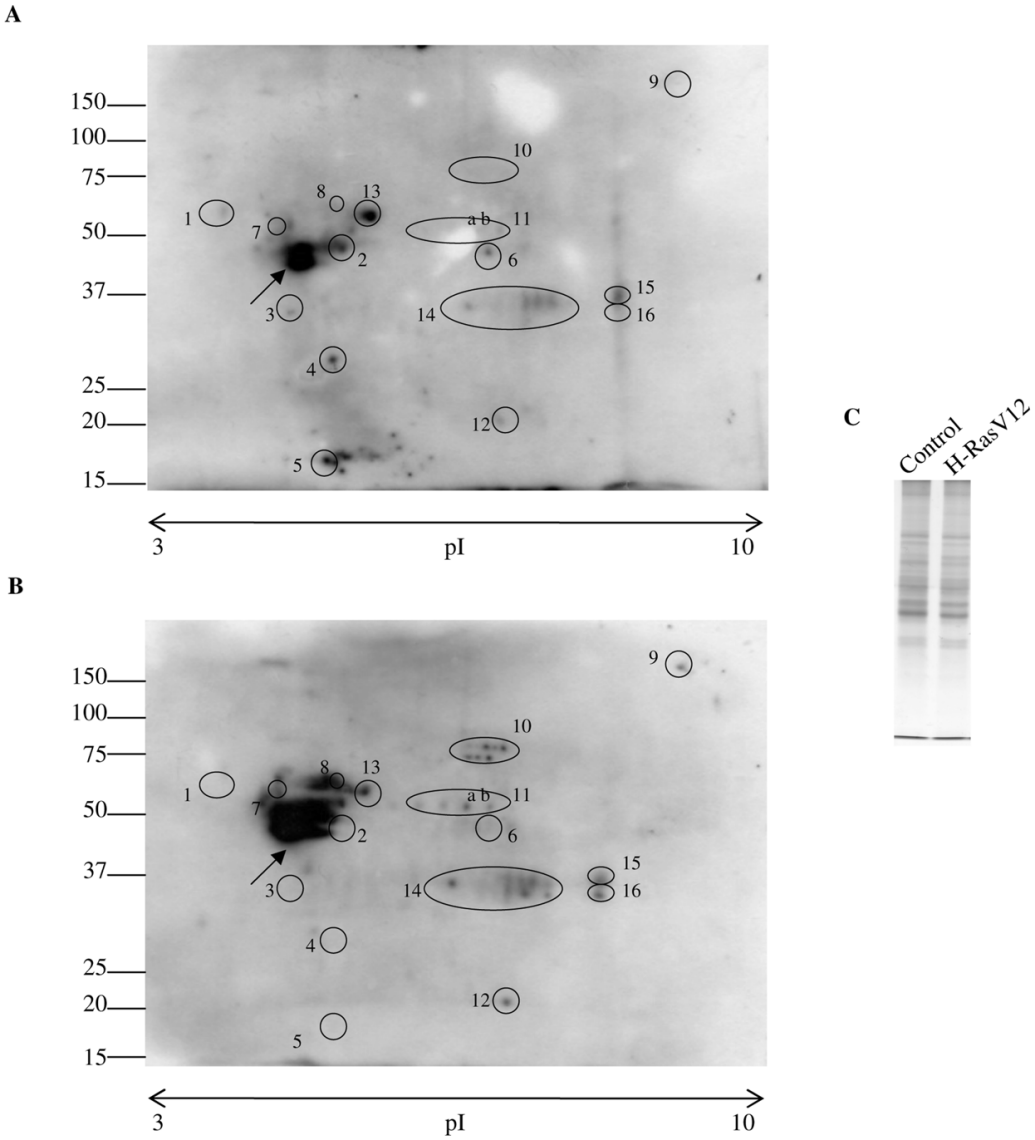
Evaluation of protein S-glutathionylation patterns. Evaluation of protein S-glutathionylation patterns was performed by 2D-immunoblotting with anti-GSH antibody: A) control cells. B) H-RasV12 expressing cells. 100 µg of total cell extracts were separated on non- linear pH 3–10 strips (7 cm) for first dimension and then loaded on 10% acrylamide/bis-acrylamide gels for second dimension. Gels were blotted on PVDF membrane. Black rounds highlight protein spots which present a different level of S-glutathionylation. C) Total loading control. A total of 20 µg of cellular extract proteins used for 2D-immunoblotting was loaded on 10% acrylamide/bis-acrylamide gel and stained with Coomassie Blu.

Due to the importance of protein S-glutathionylation in protecting proteins from oxidative stress, in signal transduction and in the modulation of protein function, we investigated protein S-glutathionylation by immunoblotting and dot blot using an anti-GSH antibody. No statistical difference of total glutathionylated proteins were observed in H-RasV12 expressing cells in comparison with the control (Fig. 5 A, B, C, D) but single bands always showed different trend levels of glutathionylated proteins in H-RasV12 expressing cells with respect to the control cells. Two-dimensional polyacrylamide gel electrophoresis (2D-PAGE), followed by western blotting analysis using the anti-GSH antibody (Fig. 6 A, B), was performed to analyse the pattern of glutathionylated proteins. Interestingly, as reported in Fig. 6, H-Ras V12 expressing cells displayed a complex pattern of protein S-glutathionylation, significantly different from their control counterparts. In particular, spots labelled from 1 to 6 were present in control cells but disappeared in H-RasV12 expressing cells, whereas spots from 7 to 12 were not detectable in control cells but were significantly S-glutathionylated in H-RasV12 expressing cells. In addition, a significant decrease of S-glutahionylation was detected for spot 13 in H-RasV12 cells, whereas an increase was detected for spots 14 and 16. Furthermore, a very intense spot (black arrow) also clearly displayed a different S-glutathionylation pattern upon H-RasV12 expression. Overall results provided strong evidence that H-RasV12 expression modified human fibroblasts S-glutathionylation pattern in a very specific manner. In order to identify the glutathionylated spots, a preparative gel was performed and stained with colloidal Coomassie. The Western blot images were matched to the corresponding colloidal Coomassie stained 2-DE gels using ImageMaster 2D-Platinum software and spots corresponding to glutathionylated proteins were cut and subjected to mass spectrometry analysis. The increment of protein load in preparative gels caused an expected loss of separative power and some spots clearly present in western blot, were not distinctly detected and resolved in Coomassie stained gels. Among the glutathyonilated proteins we were able to identify spots 7, 8 and 11a and 11b by mass spectrometry. Mass data of the spots were reported in Table 1.

**Table 1:** Mass spectrometry analysis of glutathionylated proteins.

Spot Number^a^	Accession Number^b^	Mascot Search Results^c^			MW (Da)	Calculated pI	Protein
		Matched Peptide/Searched Peptide	Sequence Coverage	Score			
7	P06576	20/34	41%	179	56525	5.26	ATP synthase subunit beta, mitochondrial
8	P08670	12/15	26%	142	53776	5.06	Vimentin
11a	P06733	16/23	38%	144	47481	7.01	Alpha enolase
11b	P06733	9/23	18%	73	47481	7.01	Alpha enolase

a Spot numbers match those reported in the representative 2-DE images shown in Fig. 6.

b Accession number in Swiss-Prot/UniprotKB.

c MASCOT search results: number of matched peptides correspond to peptide masses matching the top hit from Ms-Fit PMF, searched peptide are also reported; sequence coverage indicates [number of identified residues/total number of amino acid residues in the protein sequence]×100%; score corresponds to MASCOT score (MatrixScience, London, UK; http.//www.matrixscience.com).

### Treatment with Antioxidants Mix Induced a Transient Recovery of H-RasV12 Expressing Cells

**Figure 7:**
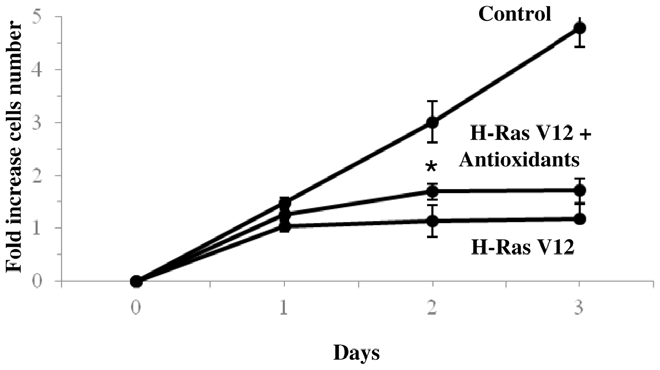
Effect of antioxidant mix on H-RasV12 cell population. Growth curve of HuDe fibroblasts transfected with H-RasV12 treated with 0.5 mM GSH ethyl ester, 50 µM α-tocopherol and 1.5 mM vitamin C. The cell number increase after the end of selection with Blasticidin-S is reported and results were compared with growth curve obtained for control and H-RasV12 transfected cells. Mean values were calculated on 5 replicates and mean ± s.d. was indicated for each sample. *P<0.05.

The treatment of H-RasV12 expressing cells with antioxidants mix resulted in a transient recovery of cell growth, as cell population increases only at a day 1 from the end of selection (Fig. 7). To not interfere with the transfection the treatment was carried out at the end of the selection. In these conditions, cells certainly suffered DNA hyper-proliferation and senescence pathways activation. Nevertheless it was possible to observe a modest recovery, of treated cells growth. This positive trend may indicate an insufficient role of antioxidant defence in the context of induced senescence.

## Discussion

Uncontrolled cell proliferation and oncogenic transformation of primary cells require oncogene deregulation and activation of the cooperating mechanism. Indeed only constitutively activated oncogene H-RasV12 is known to induce premature senescence which is considered like a first barrier to oncogenic cell progression [7], [10].In this work we report that, despite cell growth arrest and altered morphology, H-RasV12 expressing cells are actually distributed in all phases of the cell cycle, thus suggesting the activation of DNA damage checkpoints at multiple phases of cell cycle. In addition, we also observed an increase of cell death by apoptosis and autophagy. These results suggest complex dynamics of premature senescence and cell death induced by oncogene activation, whose elucidation is very important for understanding how cells escaping from proliferation barriers can acquire a progressively aberrant oncogenic potential. Previous studies showed that oncogenic H-Ras induces proliferative arrest or senescence by activating S-phase-specific DNA-damage checkpoint response (DDR) followed by apoptotic cell death [10] or that the expression of oncogenic H-Ras leads to caspase-independent cell death with autophagy features [4]. In this study we propose a role of the cellular redox balance in premature senescence and death induced from activated oncogene H-RasV12. On the other hand activated H-Ras has been shown to promote ROS production in a variety of human cancer cell types including transformed embryonic lung cells, fibroblasts, neuroblastoma cells as well as in hematopoietic progenitor cells where the promotion of superoxide and H_2_O_2_ production through activation of NADPH oxidase was shown [43]–[46]. Furthermore it has also been demonstrated that activation of H-Ras and ERK, in human renal cancer cells, promoted nuclear translocation of the transcription factor Nrf2 for its binding to the multiple sequence codified proteins link to antioxidant cellular defences [47]. Many studies have been conducted in human cancer cells but this study is the first one that has considered cellular redox balance in OIS condition activated by H-Ras.

We observed that in OIS human fibroblasts expressing H-RasV12 cellular redox imbalance was mainly caused by depletion of total antioxidant capacity rather than by a high increase in oxidant level. In fact, we did not observe high ROS production in cells over-expressing active H-Ras, but detected a remarkable reduction of antioxidant capacity. This could arise as a response to the weak but chronic oxidative stress due to DNA hyper-proliferation and/or to GSH consumption. In effect, according to the lower antioxidant capacity, we also observed a remarkable depletion of GSH, which is the cell most abundant in antioxidants and it is generally considered to influence the cell redox balance by helping to maintain a reduced intracellular environment. GSH fulfils a range of cellular functions from detoxification to protection from oxidative damage. It is also considered crucial in several cellular processes including cell differentiation, programmed cell death and cell cycle regulation [29]. Interestingly, some studies reported that cells with high proliferation rate have high GSH levels and tumor cells typically show high levels of GSH and consequently high antioxidant defenses [31]–[33]. Taken together, these data suggest that cells, which are able to maintain high levels of GSH, can escape the oncogenic barrier established following oncogene activation, whereas OIS is associated with the maintenance of low levels of antioxidant capacity.

On the contrary it was previously reported that treatment of cells with N-acetyl cysteine only marginally inhibits DNA-damage responses induced by oncogenic activation, which suggests OIS may occur independently from ROS levels [17], [48]. We showed that the treatment of active H-Ras expressing cells with antioxidants mix did not result in a significantly different cell growth compared to control cells (only a transient improvement), indicating that the treatment is not fully effective in bypassing senescence. To summarize, it was reported high levels of GSH in tumour cells [31], [33], and we observed low levels of GSH in OIS cells but the external supply of antioxidants did not radically change the induction of senescence. We can therefore assume that the level of GSH in the cell is important in the context of proliferation and that this level is adjusted internally by the cell and is independent of the availability of precursor molecules. In our study we observed depletion of GSH and also GSSG so we assumed that GSSG, produced during DNA iper-proliferation stress, could to be used for the glutathionylation of proteins. In this way a molecule of GSH could bind to cysteine residues of the protein to protect it from oxidation while the other molecule reconstituted the GSH cell levels. This supposition is also strengthened by the lower activity of glutathione reductase enzyme observed in H-Ras-expressing cells. S-glutathionylation can serve as a protective mechanism against cysteine oxidation and is recognized to be an important component of cellular redox signalling [49]–[51].

In our study we report a change in S-glutathionylation pattern in active H-RasV12 expressing cells. These findings suggest that cells may utilize GSH in post-translation protein modification thereby creating regulatory mechanisms related to cell survival. As a matter of fact, S-glutathionylation of several proteins has been reported, including many proteins involved in cell cycle control and cell death such as GAPDH, NF-kB p65, caspase-3, p53, ERK, JNK, ATP synthase, actin and others [23], [52].

In this work we identify vimentin, ATP synthase β-subunit and α-enolase, (already known to be S-glutathionylated) [53]–[55] which are more glutathionylated in H-RasV12 cells compared to the control. This modification may play a substantial role in OIS and/or oncogenic phenotypes. For example it was seen that both ATP synthase β-subunit and α-enolase are inhibited by cysteine modifications [53], [55], while there is no information about the effect on vimentin glutathionylation. Moreover it has been demonstrated that these proteins are involved both in senescence and in cancer progression phenomena [56]–[59]. The exact function of this reversible oxidative post-translational modification is unknown. Further studies investigating the specific in vivo effects of S-glutathionylation in oxidative stress are important to determine the role of S-glutathionylation during OIS and tumor progression.

Several studies have shown that many proteins can be glutathionylated or deglutathionylated during oxidative stress and in this study we demonstrate that the glutathionylation of proteins is also present during OIS. In agreement with our findings, Andrei et al. demonstrated that in some cancer cell lines proliferation inhibitors rapidly induce a substantial increase of protein S-glutathionylation levels [60], thus indicating that this event may be of relevance in the evaluation of therapeutic agents. In conclusion our results provide evidence that GSH levels decreased and protein S-glutathionylation is altered in human fibroblasts during OIS, so GSH levels and S-glutathionylation may be considered as additional barriers to oncogenic transformation.
